# Brock’s approaching zero method improved as approaching fixed point frequency to solve optimum damping ratio of dynamic vibration absorber in machine tools and experimental confirmation

**DOI:** 10.1371/journal.pone.0315289

**Published:** 2024-12-31

**Authors:** Hongliang Tian, Xixiao Liang, Xuan Du

**Affiliations:** College of Mechanical and Power Engineering, China Three Gorges University, Yichang, Hubei, The People’s Republic of China; Federal University of Technology - Parana, BRAZIL

## Abstract

The design parameters of the dynamic vibration absorber significantly affect the motion performance of the main vibration system. The Brock’s approaching zero method was improved as approaching the fixed point frequency method. A general method of obtaining the explicit exact solution to the optimum damping ratio was presented to improve the accuracy of calculating the dynamic vibration absorber’s optimum parameter. Some exact closed-form solutions, for example displacement amplitude gain, fixed point coordinate, and optimum damping ratio, were deduced with the real number form of differential equation of load motion and employing L’Hospital first rule. Many computational parameters of the main vibration system were attained. The fixed point theory essentially computes the extreme large value, not the maximum value. The numerical simulation results of the present paper’s absorber are closer to the vibrational experimental results than those of the Ormondroyd absorber and Lanchester absorber. Moreover, the present paper’s absorber has larger band width than the Ormondroyd absorber and Lanchester absorber. The current answers may be applicable to realize and control the accurate dynamic performances of the main vibration system and dynamic vibration absorber in operation.

## 1. Introduction and motivation

When researching the forced vibration of the main system with one degree of freedom, it is shown how the amplitude of this vibration can be reduced by a proper choice of the spring constant so that the main system will be far away from resonance, or by a proper balance which minimizes the magnitude of the pulsating force. Sometimes these methods are impractical therefore a particular apparatus for reducing vibrations, named the dynamic vibration absorber, must be utilized. By a proper choice of the mass, spring constant and viscous damping coefficient, a substantial reduction of the main system can be accomplished.

The past 115 years have witnessed so much international study from theory and experiment in the field of the dynamic vibration absorber, invented by Frahm since 1909. Timoshenko [1] deduced a simple formula giving the proper value of tuning the dynamic vibration absorber. Den Hartog [2] derived a very simple formula giving the correct relative tuning for each dynamic vibration absorber size. In 1928, Ormondroyd and Den Hartog presented the optimization criterion of the dynamic vibration absorber so that minimizing the maximum amplitude response of the main mass. After five years in 1933, Hahnkamm [3] derived the optimum tuning ratio of the dynamic vibration absorber from this criterion in the very first place. After thirteen years in 1946, Brock [4] directly presented fourteen formulas for optimum damping for three cases of the dynamic vibration absorber with damping and indicated the method of derivation with only one page; in the original instance, however, this writer would require twenty paragraphs of material readily available elsewhere to introduce three paragraphs of new results. At one’s earliest convenience and after only three months, Brock [5] directed attention to an error (locate citation Ref. [4, Eq (3)]) for veracity and celerity. Yu and Jia et al. [6] combined the focusing property of an acoustic black hole in a wide frequency band with the advantage of a dynamic vibration absorber. Xing and Yang [7] investigated the vibration control performance of a combined vibration isolation system consisting of a quasi-zero stiffness system and a linear dynamic vibration absorber. Du and Zou et al. [8] proposed a theoretical model of stiffened plate with multiple dynamic vibration absorbers under different boundary constraints. Sheng and He et al. [9] presented a modified add-on strategy to reduce the transverse vibration of beam structures using multiple acoustic black hole dynamic vibration absorbers. Wang and Zhou et al. [10] proposed a dual-function quasi-zero-stiffness energy harvesting dynamic vibration absorber to mitigate vibration and harvest energy simultaneously. Chen and Leng et al. [11] proposed a triple-magnet magnetic suspension dynamic vibration absorber only composed of triple cylindrical permanent magnets and an acrylic tube. Qin and Tan et al. [12] proposed an analytical model to study the effect of dynamic vibration absorbers on the vibration of a lightweight joist structure by combining the bending modes of the plate with the bending and torsional modes of the beams. Wu and Li [13] developed a data-driven method based on excitation-dependent representative basis to design the distributed dynamic vibration absorbers for the broadband vibration suppression of thin-walled structures. Chang and Zhou et al. [14] proposed a semi-active quasi-zero-stiffness dynamic vibration absorber to broaden the frequency bandwidth for ultra-low frequency vibration absorption. Li and Ikago et al. [15] proposed utilizing the inertance effect in an inerter eddy current damper to construct a dynamic vibration absorber for the seismic protection of civil structures, termed as the tuned inerter eddy current damper. Sun and Wong et al. [16] tested a tunable electromagnetic shunt damper with different opposing magnet pairs configurations for the optimum design of dynamic vibration absorber. Su and Zheng et al. [17] presented a dynamic vibration absorber with negative stiffness in controlling the first longitudinal mode of the propulsion shafting system. Lin and Zhang et al. [18] investigated the band structure, effective indices and stability of the metamaterial considering an infinite lattice. Li and Wu et al. [19] proposed a mathematical formulation for the dynamic vibration absorber design which is suitable for suppressing the structural vibration characterized by multiple natural modes. Kassem and Yang et al. [20] proposed a flutter suppression technique using an active dynamic vibration absorber. Noori and Arcos et al. [21] investigated the efficiency of dynamic vibration absorbers as a vibration abatement solution for railway-induced vibrations in the framework of a double-deck circular railway tunnel infrastructure. Beltrán-Carbajal and Silva-Navarro [22] dealt with the multi-frequency harmonic vibration suppression problem in forced Duffing mechanical systems using passive and active linear mass-spring-damper dynamic vibration absorbers. Viana and Kotinda et al. [23] dealt with the optimal tuning of two different types of dynamic vibration absorbers by using ant colony optimization, namely the vibrating blade dynamic vibration absorbers and the multi-mode dynamic vibration absorbers. Kecik [24] presented a system which consists of two main parts: an autoparametric pendulum vibration absorber and an energy harvester device, for simultaneous energy harvesting and vibration mitigation. Liu and Liu [25] applied Brock’s approach to a different type of damped vibration absorber. Ren [26] proposed a layout of a dynamic vibration absorber and developed its optimum design principles.

Until nowadays, the exact parameter optimization method of the dynamic vibration absorber is not studied fully. Three concrete shortcomings of study results on the dynamic vibration absorber are as follows: (1) the real number force *F* sin *ωt* and complex number force *F*e^*iωt*^ are not distinguished, and the complex number response displacement and real number response displacement amplitude are not distinguished, too; (2) all amplitude-frequency response characteristic pass through some fixed points independent of the damping ratio, however these fixed points are the extreme large value points not the required maximum value points; and (3) to the best of our knowledge, unfortunately there is not a general and convenient method attaining the exact answer to the optimum damping ratio of the dynamic vibration absorber.

A general method of obtaining the explicit exact answer to the optimum damping ratio of the dynamic vibration absorber was presented. The damping element is not connected to the main system to be controlled, but to the skyhook. The skyhook damper is perceived as an ideal active suspension device. Many exact analytic solutions, such as displacement amplitude gain, and fixed point coordinate, were deduced in minute detail. The optimum damping ratio of the dynamic vibration absorber was attained by approaching the fixed point frequency method. Some computational parameters of the main vibration system were given. The proposed absorber is compared with those already known such as the Ormondroyd absorber [2] and Lanchester absorber [1]. For the same mass ratio, this design of the proposed damper can offer a more effective control result over an ordinary design. The proposed damper has larger band width. The proposed model can bring convenience in practice in the automobile, sports equipment and computerized numerical control lathe.

## 2. Displacement amplitude gain of main mass

**Fig 1:**
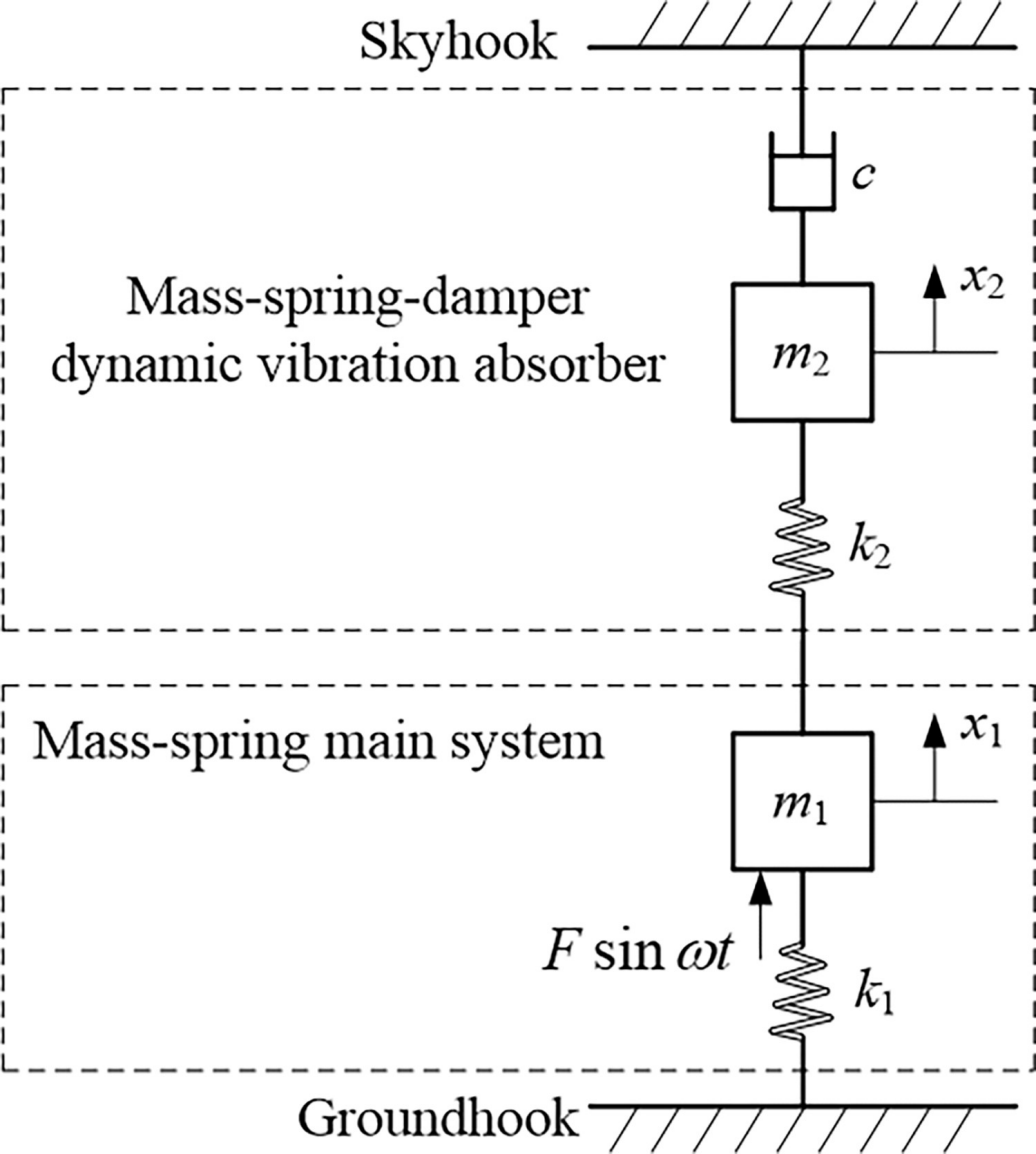
Mass-spring-damper dynamic vibration absorber attached to mass-spring main system.

In Fig 1 let the combination *m*_1_, *k*_1_ denote the schematic representation of the main system under consideration, with a pulsating force *F* sin *ωt* acting on it, where *m*_1_ is called the main mass, and *k*_1_ the main groundhook stiffness. The dynamic vibration absorber consists of a comparatively small vibratory system *m*_2_, *k*_2_, *c*, where *m*_2_ is called the attached mass, *k*_2_ the connection stiffness between two masses *m*_1_ and *m*_2_, and *c* the attached skyhook damping.

The quantity *c* is known as the damping constant or more at length as the coefficient of viscous damping. The motions of the main system and the dynamic vibration absorber are governed by the two-order differential equation of load motion in the two order matrix form

$$(\begin{array}{cc} m_{1} & 0\\ 0 & m_{2} \end{array})(\begin{array}{l} \frac{d^{2}x_{1}}{dt^{2}}\\ \frac{d^{2}x_{2}}{dt^{2}} \end{array})+(\begin{array}{cc} 0 & 0\\ 0 & c \end{array})(\begin{array}{l} \frac{dx_{1}}{dt}\\ \frac{dx_{2}}{dt} \end{array})+(\begin{array}{cc} k_{1}+k_{2} & -k_{2}\\ -k_{2} & k_{2} \end{array})(\begin{array}{l} x_{1}\\ x_{2} \end{array})=(\begin{array}{l} F\\ 0 \end{array})\sin\;\omega t$$ (1)

where *x*_1_ and *x*_2_ separately denote the displacement of masses *m*_1_ and *m*_2_, *F* the amplitude of sinusoidal harmonic disturbing force, *ω* the forced angular frequency, and *t* the time.

Eq (1) is the imaginary part of the following equation

$$(\begin{array}{cc} m_{1} & 0\\ 0 & m_{2} \end{array})(\begin{array}{l} \frac{d^{2}X_{1}}{dt^{2}}\\ \frac{d^{2}X_{2}}{dt^{2}} \end{array})+(\begin{array}{cc} 0 & 0\\ 0 & c \end{array})(\begin{array}{l} \frac{dX_{1}}{dt}\\ \frac{dX_{2}}{dt} \end{array})+(\begin{array}{cc} k_{1}+k_{2} & -k_{2}\\ -k_{2} & k_{2} \end{array})(\begin{array}{l} X_{1}\\ X_{2} \end{array})=(\begin{array}{l} F\\ 0 \end{array})e^{i\omega t}$$ (2)

where the small letter e is the natural constant, and the Roman $i=\sqrt{-1}$ sometimes called the imaginary number unit. So *x*_1_ = Im *X*_1_, *x*_2_ = Im *X*_2_.

A particular solution to Eq (2) may be written as

$$\begin{array}{l} X_{1}={\hat{X}}_{1}e^{i\omega t}\\ X_{2}={\hat{X}}_{2}e^{i\omega t} \end{array}\}$$ (3)

Substituting Eq (3) into Eq (2) results in

$$\left(\begin{array}{cc} k_{1}+k_{2}-m_{1}\omega^{2} & -k_{2}\\ -k_{2} & k_{2}-m_{2}\omega^{2}+ic\omega \end{array})(\begin{array}{l} {\hat{X}}_{1}\\ {\hat{X}}_{2} \end{array})=(\begin{array}{l} F\\ 0 \end{array}\right)$$ (4)

$$\left(\begin{array}{cc} 1+\frac{k_{2}}{k_{1}}-\frac{m_{1}\omega^{2}}{k_{1}} & -\frac{k_{2}}{k_{1}}\\ -\frac{k_{2}}{k_{1}} & \frac{k_{2}}{k_{1}}-\frac{m_{2}\omega^{2}}{k_{1}}+i\frac{c\omega }{k_{1}} \end{array})(\begin{array}{l} {\hat{X}}_{1}\\ {\hat{X}}_{2} \end{array})=(\begin{array}{l} \frac{F}{k_{1}}\\ 0 \end{array}\right)$$ (5)

After performing much algebra Eq (5) is transformed into

$$\left(\begin{array}{l} {\hat{X}}_{1}\\ {\hat{X}}_{2} \end{array})={\left(\begin{array}{cc} 1+\mu f^{2}-g^{2} & -\mu f^{2}\\ -\mu f^{2} & \mu f^{2}-\mu g^{2}+i2\mu f\zeta g \end{array}\right)}^{-1}(\begin{array}{l} \frac{F}{k_{1}}\\ 0 \end{array}\right)$$ (6)

where $\mu =\frac{m_{2}}{m_{1}}$ stands for the mass ratio; $f=\frac{\omega_{2}}{\omega_{1}}$ is the tuning ratio, herein $\omega_{2}=\sqrt{\frac{k_{2}}{m_{2}}}$ the natural angular frequency of dynamic vibration absorber, and $\omega_{1}=\sqrt{\frac{k_{1}}{m_{1}}}$ the natural angular frequency of main system; $\zeta =\frac{c}{2m_{2}\omega_{2}}$ represents the damping ratio of dynamic vibration absorber; and $g=\frac{\omega }{\omega_{1}}$ is the forced angular frequency ratio. Four dimensionless variables of *μ* (determining *m*_2_), *f* (*m*_2_ having been determined, thus determining *k*_2_), *ζ* (determining *c*), and *g* (determining *ω*) are mutually independent.

Solving Eq (6) yields

$$\left(\begin{array}{l} {\hat{X}}_{1}\\ {\hat{X}}_{2} \end{array})=\frac{\frac{F}{k_{1}}}{\left(g^{2}-1)(g^{2}-f^{2})-\mu f^{2}g^{2}+i2f\zeta g(1+\mu f^{2}-g^{2}\right)}(\begin{array}{c} f^{2}-g^{2}+i2f\zeta g\\ f^{2} \end{array}\right)$$ (7)

Therefore there exists an algebraic form

$${\hat{X}}_{1}=\frac{F}{k_{1}}\cdot \frac{f^{2}-g^{2}+i2f\zeta g}{\left(g^{2}-1)(g^{2}-f^{2})-\mu f^{2}g^{2}+i2f\zeta g(1+\mu f^{2}-g^{2}\right)}=|{\hat{X}}_{1}|e^{i\varphi_{1}}$$ (8)

Hence $X_{1}=|{\hat{X}}_{1}|e^{i\varphi_{1}}e^{i\omega t}=|{\hat{X}}_{1}|e^{i(\omega t+\varphi_{1})}=|X_{1}|e^{i(\omega t+\varphi_{1})}$, $x_{1}=|X_{1}|\sin(\omega t+\varphi_{1})$.

Four-parameter universal displacement amplitude gain for the steady-state response of the main mass is defined by

$$A_{1}=\frac{\left|{\hat{X}}_{1}\right|}{F/k_{1}}=\left|\frac{f^{2}-g^{2}+i2f\zeta g}{\left(g^{2}-1)(g^{2}-f^{2})-\mu f^{2}g^{2}+i2f\zeta g(1+\mu f^{2}-g^{2}\right)}\right|=\sqrt{\frac{{\left(f^{2}-g^{2}\right)}^{2}+4f^{2}\zeta^{2}g^{2}}{{\left[(g^{2}-1)(g^{2}-f^{2})-\mu f^{2}g^{2}\right]}^{2}+4f^{2}\zeta^{2}g^{2}{\left(1+\mu f^{2}-g^{2}\right)}^{2}}}$$ (9)

## 3. Conditions for equal ordinates of two fixed points of main system

### 3.1 Two fixed points of main system independent of damping ratio

Nowadays so that Eq (9) will be independent of *ζ* only if one acquires

$$A_{1}^{2}={\left[\frac{f^{2}-g^{2}}{(g^{2}-1)(g^{2}-f^{2})-\mu f^{2}g^{2}}\right]}^{2}={\left(\frac{1}{1+\mu f^{2}-g^{2}}\right)}^{2}$$ (10)

We can obliterate the square sign on both sides but then have to add a ± in front of the right side under this condition as

$$\frac{f^{2}-g^{2}}{(g^{2}-1)(g^{2}-f^{2})-\mu f^{2}g^{2}}=\pm \frac{1}{1+\mu f^{2}-g^{2}}$$ (11)

With the plus sign, therefore Eq (11) becomes

$$\frac{f^{2}-g^{2}}{(g^{2}-1)(g^{2}-f^{2})-\mu f^{2}g^{2}}=\frac{1}{1+\mu f^{2}-g^{2}}$$ (12)

Cross-multiplication later, one has

$$\mu f^{4}=0$$ (13)

Nevertheless, as *μ* > 0, *f* > 0, there is no answer to Eq (13).

The other alternative is the minus sign before the right side of Eq (11)

$$\frac{f^{2}-g^{2}}{(g^{2}-1)(g^{2}-f^{2})-\mu f^{2}g^{2}}=-\frac{1}{1+\mu f^{2}-g^{2}}$$ (14)

A short calculation later Eq (14) then reads as follows

$$\frac{\left(g^{2}-1)(g^{2}-f^{2})-\mu f^{2}g^{2}+(f^{2}-g^{2})(1+\mu f^{2}-g^{2}\right)}{2}=g^{4}-(1+f^{2}+\mu f^{2})g^{2}+f^{2}+\frac{\mu f^{4}}{2}=0$$ (15)

From this equation two roots $g_{S}^{2}$ and $g_{T}^{2}$ can be found which determine the abscissas of the points *S* and *T*. The root discriminant of the quadratic equation in one unknown (15) is defined as

$$\Delta ={\left[1+(1+\mu )f^{2}\right]}^{2}-4f^{2}-2\mu f^{4}$$ (16)

Eq (16) can be expanded

$$\Delta =1+2(1+\mu )f^{2}+(1+2\mu +\mu^{2})f^{4}-4f^{2}-2\mu f^{4}$$ (17)

Afterwards Eq (17) can be reduced to the form

$$\Delta =1+2(\mu -1)f^{2}+(1+\mu^{2})f^{4}$$ (18)

At last the discriminant can be deduced as

$$\Delta ={\left(1+\mu f^{2}-f^{2}\right)}^{2}+2\mu f^{4}$$ (19)

According to the extracting root formula of quadratic equation in one unknown, the abscissas of fixed points *S* and *T* of the (*g*, *A*_1_) diagram are, respectively

$$g_{S}=\sqrt{\frac{1+f^{2}+\mu f^{2}-\sqrt{{\left(1+\mu f^{2}-f^{2}\right)}^{2}+2\mu f^{4}}}{2}}$$ (20)

$$g_{T}=\sqrt{\frac{1+f^{2}+\mu f^{2}+\sqrt{{\left(1+\mu f^{2}-f^{2}\right)}^{2}+2\mu f^{4}}}{2}}$$ (21)

By the aid of Eq (15), an auxiliary function is constructed

$$\psi (g)=g^{4}-(1+f^{2}+\mu f^{2})g^{2}+f^{2}+\frac{\mu f^{4}}{2}$$ (22)

Taking Eqs (22), (15), (20) and (21) produces

$$\psi (0)=f^{2}+\frac{\mu f^{4}}{2}>0,\;\psi (g_{S})=\psi (g_{T})=0$$ (23)

The critical frequency is obtained by equating the denominator of the right side of Eq (11) to zero, obviously $g_{\mathrm{cr}}=\sqrt{1+\mu f^{2}}$, and

$$\psi (\sqrt{1+\mu f^{2}})={\left(1+\mu f^{2}\right)}^{2}-(1+f^{2}+\mu f^{2})(1+\mu f^{2})+f^{2}+\frac{\mu f^{4}}{2}$$ (24)

Then Eq (24) can be put into the form

$$\psi (\sqrt{1+\mu f^{2}})=-\frac{\mu f^{4}}{2}<0$$ (25)

Making use of Eqs (15), (23), (25) and the open upward property of parabola, one obtains

$$0<g_{S}<\sqrt{1+\mu f^{2}}<g_{T}$$ (26)

The corresponding values of the amplitudes of the forced vibration are obtained by introducing Eqs (20) and (21) in Eq (9) or in the right side of Eq (10) or (14). Using the latter as a simpler one, the ordinates of points *S* and *T* are determined by, respectively

$$A_{1S}=\frac{1}{1+\mu f^{2}-g_{S}^{2}}$$ (27)

$$A_{1T}=\frac{1}{g_{T}^{2}-1-\mu f^{2}}$$ (28)

### 3.2 Equal ordinates of two fixed points *S* and *T* of main system

Through Eqs (20) and (21), we have (or the sum of two roots of quadratic equation in one unknown (15))

$$g_{S}^{2}+g_{T}^{2}=1+f^{2}+\mu f^{2}$$ (29)

By changing the tuning ratio *f*, two fixed points *S* and *T* can be shifted up and down the curve for *ζ* = 0. By changing *f*, one point goes up and the other down. It is reasonable to expect that the most favorable condition will be obtained by making the ordinates of points *S* and *T* equal. In order to reach the optimum tuning ratio, this requires that

$$A_{1S}=A_{1T}$$ (30)

Substituting this condition (30) in Eqs (27) and (28) stands

$$g_{S}^{2}+g_{T}^{2}=2+2\mu f^{2}$$ (31)

Eq (29) is equal to Eq (31) with the optimum tuning ratio under a condition

$$f=\frac{1}{\sqrt{1-\mu }}\;(\mu <1)$$ (32)

Inserting Eq (32) into Eqs (20) and (21), respectively, brings

$$g_{S}=\sqrt{\frac{1-\sqrt{\frac{\mu }{2}}}{1-\mu }}\;(0<\mu <1)$$ (33)

$$g_{T}=\sqrt{\frac{1+\sqrt{\frac{\mu }{2}}}{1-\mu }}\;(0<\mu <1)$$ (34)

Inserting Eqs (32) and (33) into Eq (27) and inserting Eqs (34) and (32) into Eq (28) get the equal extreme large value

$$A_{1S}=A_{1T}=(1-\mu )\sqrt{\frac{2}{\mu }}\;(0<\mu <1)$$ (35)

## 4. Optimum damping ratio of dynamic vibration absorber

### 4.1 Brock’s approaching zero method improved as approaching fixed point frequency

Brock [4] gave the abscissa of point *P* of the diagram

$$g_{1}=\sqrt{\frac{1-\sqrt{\frac{\mu }{2+\mu }}}{1+\mu }}$$ (36)

In order to cause the curve to pass horizontally through point *P*, Brock [4] first required that it should pass through a point *P*′ of abscissa

$$g=\sqrt{\frac{1-\sqrt{\frac{\mu }{2+\mu }}+\delta }{1+\mu }}=\sqrt{\frac{1-\sqrt{\frac{\mu }{2+\mu }}}{1+\mu }+\frac{\delta }{1+\mu }}$$ (37)

And then let *δ* approach zero as a limit. Inserting Eq (36) into Eq (37) brings about

$$g=\sqrt{g_{1}^{2}+\frac{\delta }{1+\mu }}\Rightarrow \delta =(1+\mu )(g^{2}-g_{1}^{2})$$ (38)

Brock [4] obtained a result of the form

$${\left(\frac{c}{c_{0}}\right)}^{2}=\frac{A_{0}+A_{1}\delta +A_{2}\delta^{2}+A_{3}\delta^{3}+\cdots }{B_{0}+B_{1}\delta +B_{2}\delta^{2}+B_{3}\delta^{3}+\cdots }$$ (39)

Inserting Eq (38) into Eq (39) brings about

$${\left(\frac{c}{c_{0}}\right)}^{2}=\frac{\varphi (g)}{F(g)}$$ (40)

Since Eq (40) assumes the indeterminate form 0/0 if *δ* = 0 or *g* = *g*_1_, it is clear that our desired result is given by approaching fixed point frequency

$${\left(\frac{c}{c_{0}}\right)}^{2}=\underset{g\to g_{1}}{\lim}\frac{\varphi (g)}{F(g)}$$ (41)

Nowadays, restart from Eq (9)

$$4f^{2}\zeta^{2}g^{2}=\frac{{\left(f^{2}-g^{2}\right)}^{2}-A_{1}^{2}{\left[(g^{2}-1)(g^{2}-f^{2})-\mu f^{2}g^{2}\right]}^{2}}{A_{1}^{2}{\left(1+\mu f^{2}-g^{2}\right)}^{2}-1}$$ (42)

Based upon the formula for the difference of squares, Eq (42) can be written as

$$4f^{2}g^{2}\zeta^{2}=\frac{f^{2}-g^{2}+A_{1}[(g^{2}-1)(g^{2}-f^{2})-\mu f^{2}g^{2}]}{A_{1}(1+\mu f^{2}-g^{2})+1}\cdot \frac{f^{2}-g^{2}-A_{1}[(g^{2}-1)(g^{2}-f^{2})-\mu f^{2}g^{2}]}{A_{1}(1+\mu f^{2}-g^{2})-1}$$ (43)

In terms of Eq (43), the damping coefficient *ζ* will be optimized for the given forced angular frequency ratio *g*. A detailed explanation is associated with L’Hospital first rule in the following Sections 4.2 and 4.3.

### 4.2 Optimum damping ratio at fixed point *S*

Introducing Eq (27) into Eq (14) leads to

$$A_{1}[(g^{2}-1)(g^{2}-f^{2})-\mu f^{2}g^{2}]=g^{2}-f^{2}$$ (44)

We deduce from Eq (27)

$$A_{1}(1+\mu f^{2}-g^{2})=1$$ (45)

Introducing Eqs (45) and (44) into Eq (43) leads to

$$4f^{2}g^{2}\zeta^{2}=(f^{2}-g^{2})\frac{f^{2}-g^{2}+A_{1}[(g^{2}-1)(g^{2}-f^{2})-\mu f^{2}g^{2}]}{A_{1}(1+\mu f^{2}-g^{2})-1}$$ (46)

L’Hospital (or L’Hôpital in French) rule is divided into L’Hospital first rule suitable to solve the indeterminate form 0/0 and L’Hospital second rule suitable to solve the indeterminate form ∞/∞. On the basis of Eqs (44) and (45), as *g*→*g*_*s*_, both the numerator and denominator of the right side of Eq (46) approach zero, and for convenience, this indeterminate expression must be evaluated by using L’Hospital first rule. Adopting Eq (41), the limit form of Eq (46) can be rewritten as

$$4f^{2}g^{2}\zeta^{2}=\underset{g\to g_{S}}{\lim}(f^{2}-g^{2})\frac{g^{2}-f^{2}-A_{1}[(g^{2}-1)(g^{2}-f^{2})-\mu f^{2}g^{2}]}{A_{1}(g^{2}-1-\mu f^{2})+1}$$ (47)

According to L’Hospital first rule, the numerator and denominator of the right side of Eq (47) should be separately differentiated with respect to *g* to yield the limit form of this equation as

$$\zeta^{2}=\underset{g\to g_{S}}{\lim}\frac{1}{4}(\frac{1}{g^{2}}-\frac{1}{f^{2}})(\frac{1}{A_{1}}+1+f^{2}+\mu f^{2}-2g^{2})$$ (48)

Substitute Eqs (33), (32) and (35) into Eq (48). This is a long and important work which results in

$$\zeta^{2}=\frac{1}{4}(\frac{1-\mu }{1-\sqrt{\frac{\mu }{2}}}-1+\mu )\frac{3\sqrt{\frac{\mu }{2}}}{1-\mu }\;(0<\mu <1)$$ (49)

Rationalizing the result, the optimum damping ratio at fixed point *S* is formulated by

$$\zeta_{S}=\sqrt{\frac{3\mu }{4(2-\mu )}(1+\sqrt{\frac{\mu }{2}})}\;(0<\mu <1)$$ (50)

When *ζ* = *ζ*_*s*_, the fixed point *S* has the extreme large value but by no manner of means the maximum value.

### 4.3 Optimum damping ratio at fixed point *T*

Introducing Eq (28) into Eq (14) leads to

$$A_{1}[(g^{2}-1)(g^{2}-f^{2})-\mu f^{2}g^{2}]=f^{2}-g^{2}$$ (51)

We deduce from Eq (28)

$$A_{1}(1+\mu f^{2}-g^{2})=-1$$ (52)

Introducing Eqs (51) and (52) into Eq (43) leads to

$$4f^{2}g^{2}\zeta^{2}=(g^{2}-f^{2})\frac{f^{2}-g^{2}-A_{1}[(g^{2}-1)(g^{2}-f^{2})-\mu f^{2}g^{2}]}{A_{1}(1+\mu f^{2}-g^{2})+1}$$ (53)

Utilizing Eq (41), the limit form of Eq (53) can be rewritten as

$$4f^{2}g^{2}\zeta^{2}=\underset{g\to g_{T}}{\lim}(g^{2}-f^{2})\frac{g^{2}-f^{2}+A_{1}[(g^{2}-1)(g^{2}-f^{2})-\mu f^{2}g^{2}]}{A_{1}(g^{2}-1-\mu f^{2})-1}$$ (54)

According to L’Hospital first rule, the numerator and denominator of the right side of Eq (54) should be separately differentiated with respect to *g* to yield the limit form of this equation as

$$\zeta^{2}=\underset{g\to g_{T}}{\lim}\frac{1}{4}(\frac{1}{f^{2}}-\frac{1}{g^{2}})(\frac{1}{A_{1}}+2g^{2}-1-f^{2}-\mu f^{2})$$ (55)

Substitute Eqs (32), (34) and (35) into Eq (55). This is a long and exciting job which results in

$$\zeta^{2}=\frac{1}{4}(1-\mu -\frac{1-\mu }{1+\sqrt{\frac{\mu }{2}}})\frac{3\sqrt{\frac{\mu }{2}}}{1-\mu }\;(0<\mu <1)$$ (56)

Rationalizing the result, we obtain the optimum damping ratio at fixed point *T*

$$\zeta_{T}=\sqrt{\frac{3\mu }{4(2-\mu )}(1-\sqrt{\frac{\mu }{2}})}\;(0<\mu <1)$$ (57)

When *ζ* = *ζ*_*T*_, the fixed point *T* has the extreme large value but by no manner of means the maximum value.

## 5. Characteristics of main system and experimental confirmation

**Fig 2:**
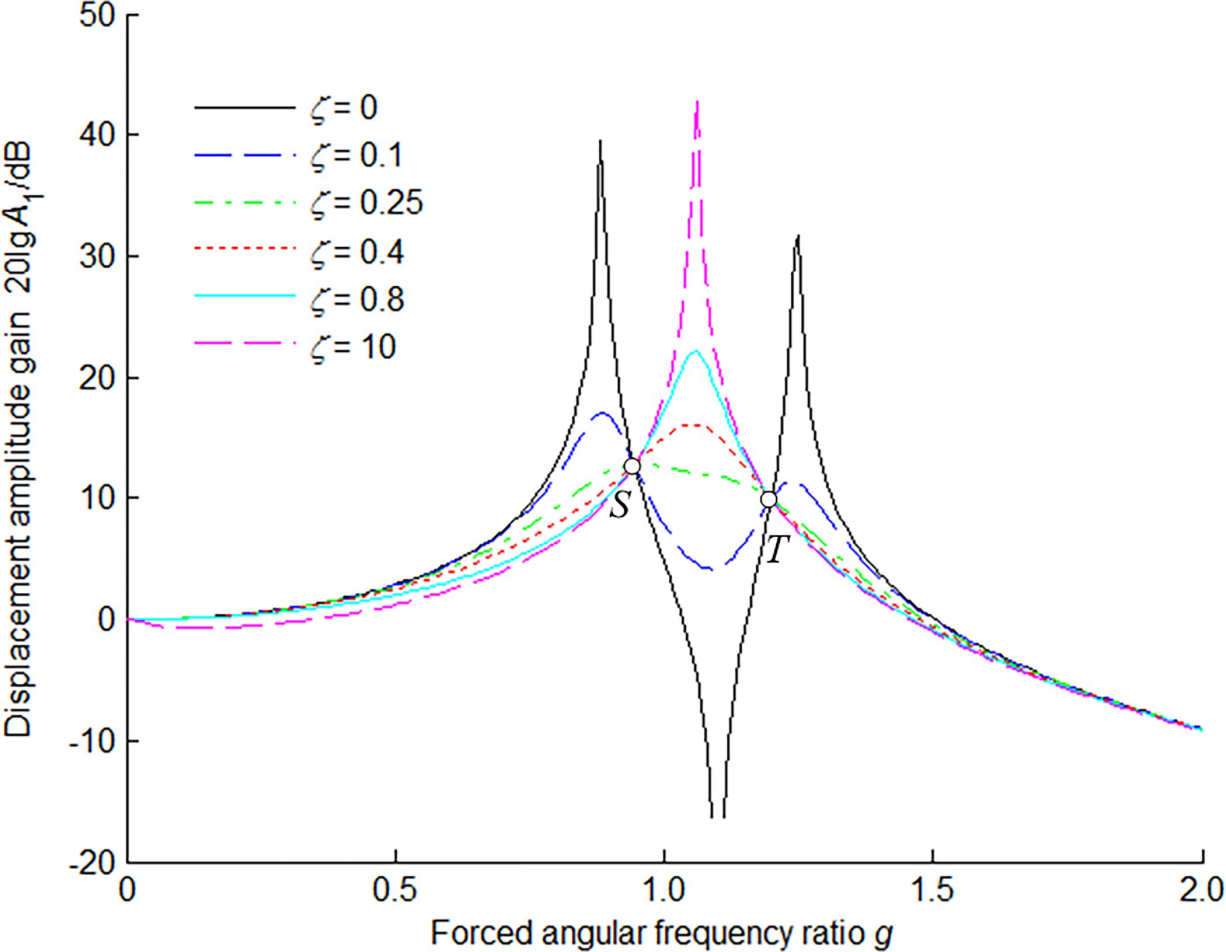
Amplitude-frequency response characteristic of main mass for *μ* = 0.1 and *f* = 1.1.

According to Eq (9), the amplitude-frequency (the relation between the displacement amplitude gain *A*_1_ and the forced angular frequency ratio *g*) response characteristic of the main mass is shown in Fig 2 for *μ* = 0.1 and *f* = 1.1 in six cases of damping ratios *ζ*. It is clearly seen that there exist two common points *S* and *T* on all the curves, where the responses are not influenced by the damping ratio. These two points are referred to as the fixed points where all the curves pass through for any damping ratio *ζ*.

**Fig 3:**
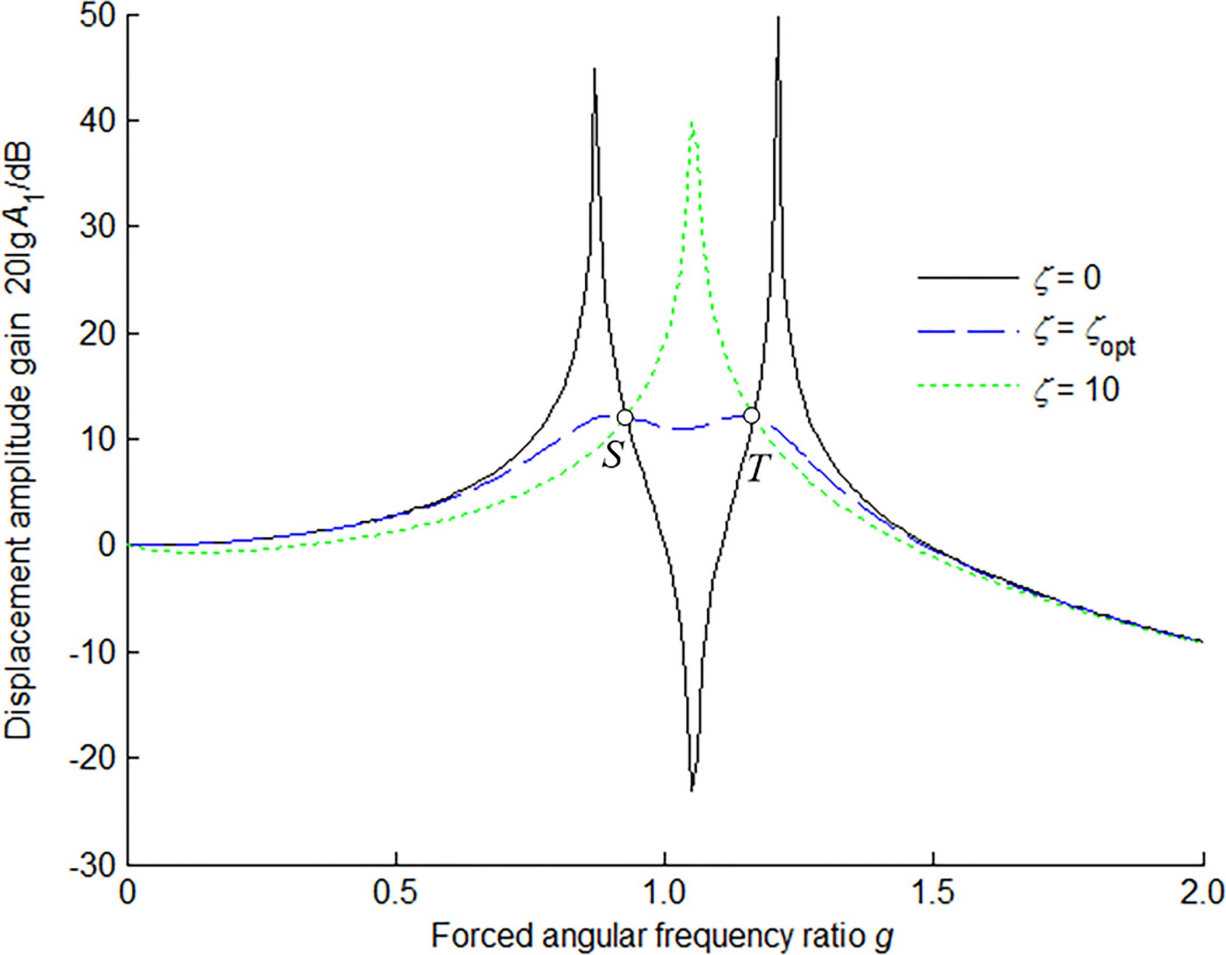
Amplitude-frequency response characteristic of main mass for *μ* = 0.1 and optimum tuning ratio.

The amplitude-frequency response characteristic of the main mass is shown in Fig 3 for *μ* = 0.1 and the optimum tuning ratio in three kinds of damping ratios. The optimum condition of the main mass can be achieved by adjusting the responses at points *S* and *T* to the same level herein, and meanwhile making points *S* and *T* the extreme large value points under the optimum damping ratio.

**Fig 4:**
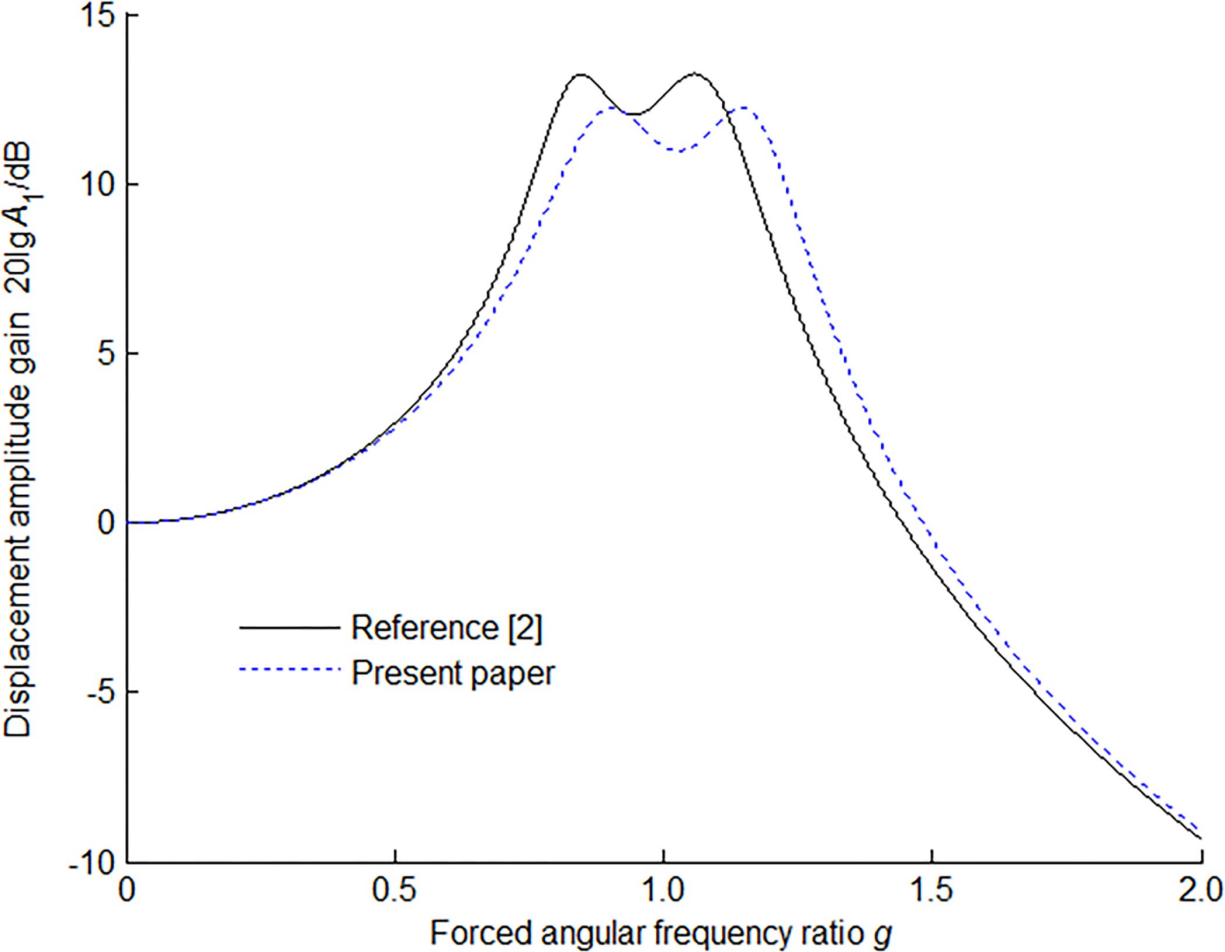
Amplitude-frequency response characteristic of main mass with optimum absorber for *μ* = 0.1.

The amplitude-frequency response characteristic of the main mass is shown in Fig 4 for *μ* = 0.1, the optimum tuning ratio and optimum damping ratio. In Fig 4 is shown the difference of the amplitude response of the main system with an optimally tuned ordinary dynamic vibration absorber and the present dynamic vibration absorber under the same mass ratio. For the same mass ratio, it can be seen that the maximum displacement amplitude gain of the present model will give a lower vibration level than that of the ordinary model.

**Fig 5:**
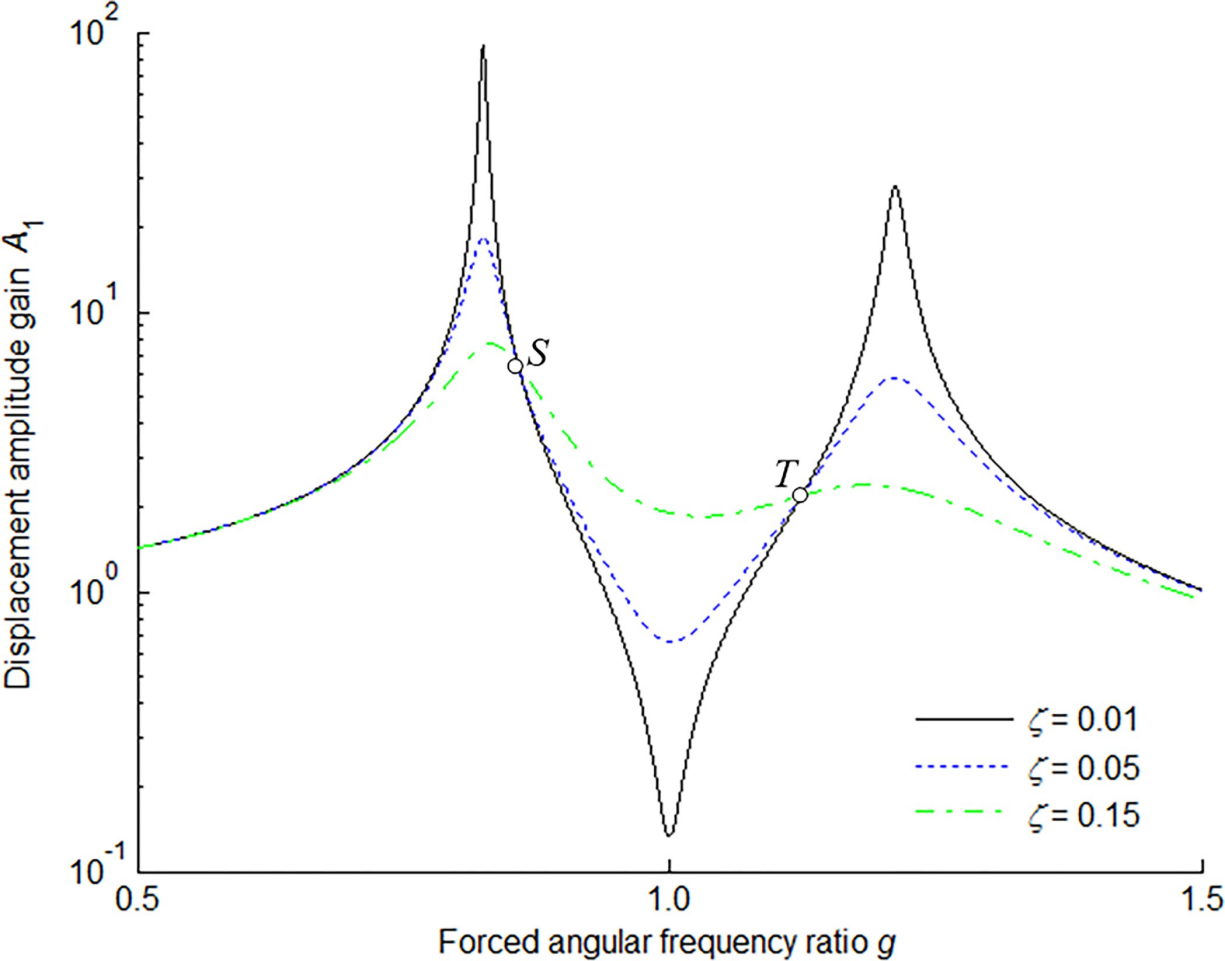
Amplitude-frequency response characteristic of main mass with Den Hartog absorber.

Using the following values: *m*_1_ = 1 kg, *k*_1_ = 8900 N/m, *m*_2_ = 0.15 kg, *f* = 1, Ref. [2] shows the amplitude-frequency response characteristic of the main mass in Fig 5. Den Hartog indicated that the optimum damping ratio must be a value for which the curve passes horizontally through either point *S* or *T*.

**Fig 6:**
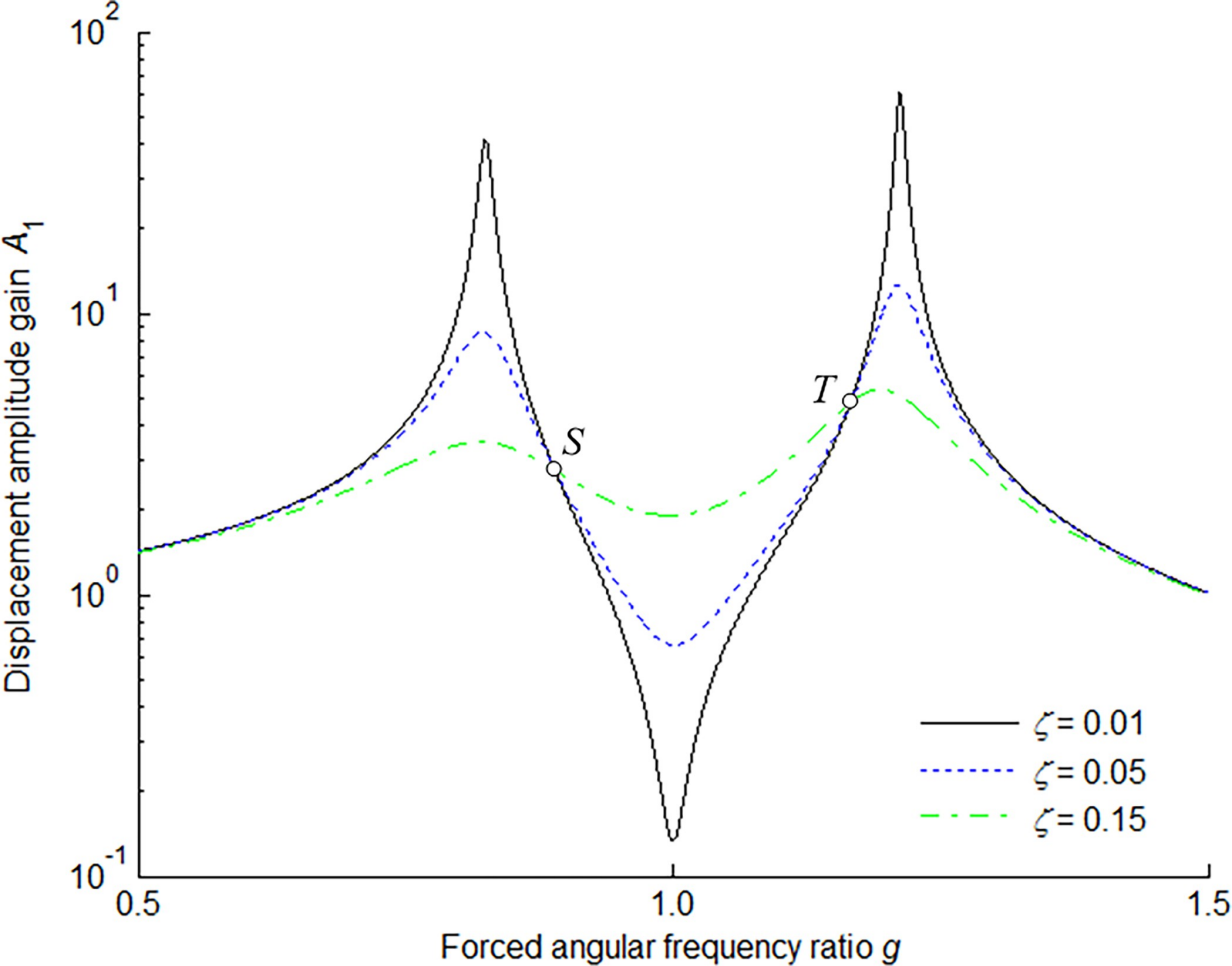
Amplitude-frequency response characteristic of main mass with present absorber.

Using the following values: *m*_1_ = 1 kg, *k*_1_ = 8900 N/m, *m*_2_ = 0.15 kg, *f* = 1, the present paper evaluates the amplitude-frequency response characteristic of the main mass in Fig 6.

**Fig 7:**
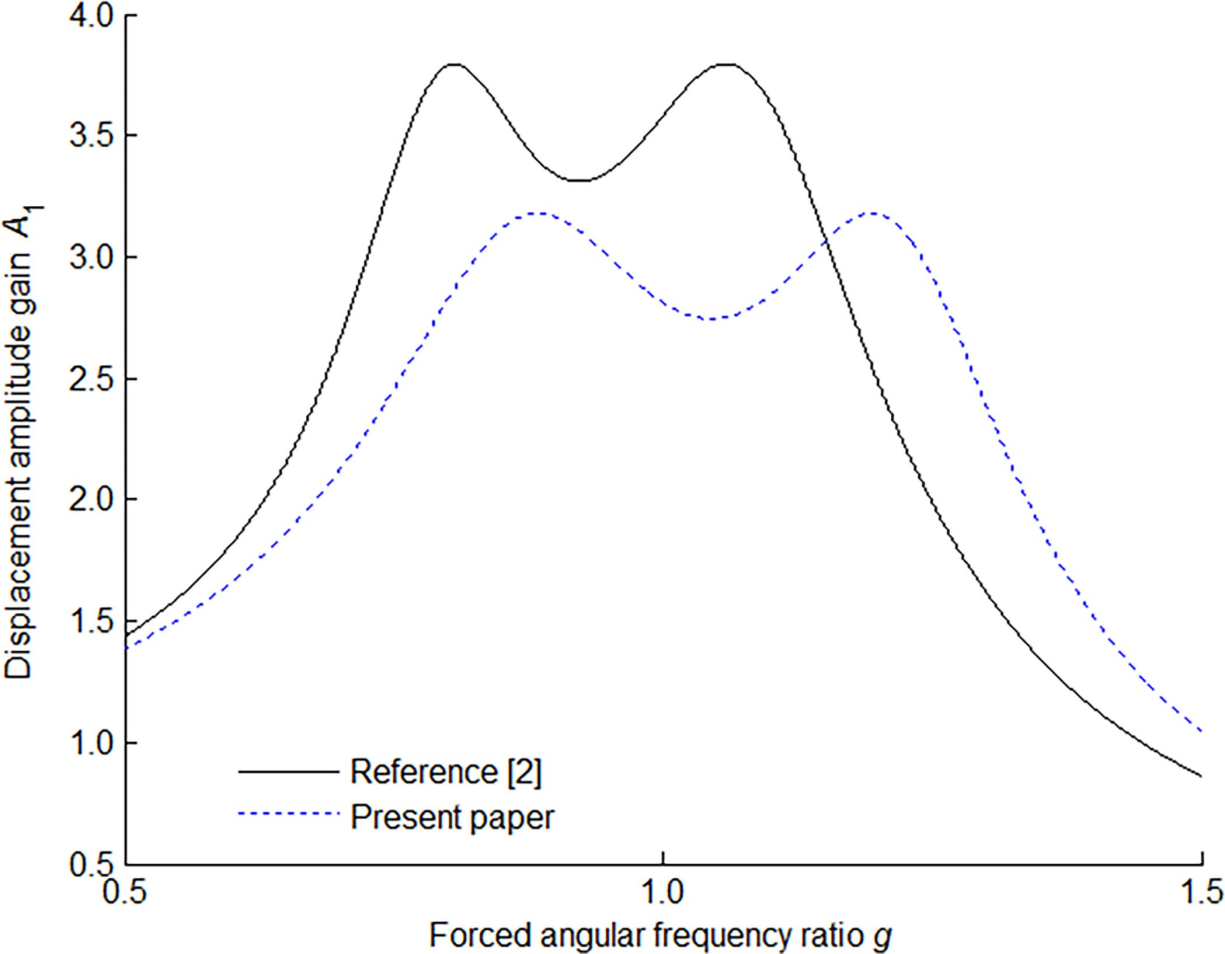
Amplitude-frequency response characteristic of main mass with optimum absorber for *μ* = 0.15.

Using the following values: *m*_1_ = 1 kg, *k*_1_ = 8900 N/m, *m*_2_ = 0.15 kg, Ref. [2] and the present paper evaluate the amplitude-frequency response characteristic of the main mass in Fig 7 and the corresponding data are listed in Table 1. In the table, utilizing the mean value theorem for definite integrals, *A*_1_(*ξ*) is the average value of displacement amplitude gain *A*_1_(*g*) on the interval [*a*, *b*] defined as

$$A_{1}(\xi )=\frac{1}{b-a}\int_{a}^{b}A_{1}(g)dg\;(a<\xi <b)$$ (58)

**Table 1:** Comparison of the two absorbers.

Absorber	*f*_opt_	*ζ*_opt_	*g*_*S*_	*g*_*T*_	*A*_1_(*g*_*S*_)	*A*_1_(*g*_*T*_)	*A*_1_(*f*_opt_)	*A*_1_(*ξ*)
Ref. [2]	0.8696	0.2212	0.7999	1.0484	3.7859	3.7859	3.4758	2.5238
Present	1.0847	0.2466	0.9243	1.2242	3.1038	3.1038	2.7949	2.3776

Employing the above-mentioned values, numerically some important data are summarized in Table 2. In the table, *A*_1_(*g*_c1_) and *A*_1_(*g*_c2_) are the crest values and their corresponding forced angular frequency ratios *g*_c1_ and *g*_c2_. *A*_1_(*g*_t_) is the trough value between the two crests and its corresponding forced angular frequency ratio *g*_t_.

**Table 2:** Some pivotal data from Fig 7 of the two absorbers.

Absorber	*A*_1_(*g*_c1_)	*g*_c1_	*A*_1_(*g*_c2_)	*g*_c2_	*A*_1_(*g*_t_)	*g*_t_
Ref. [2]	3.7927	0.8068	3.7954	1.0593	3.3114	0.9239
Present	3.1796	0.8854	3.1792	1.1958	2.7449	1.0477

The following observations can be made from Tables 1 and 2. The two crests in each of the curves are almost equal in height, that is to say, as expected, *A*_1_(*g*_*S*_) = *A*_1_(*g*_*T*_) ≈ *A*_1_(*g*_c1_) ≈ *A*_1_(*g*_c2_). For the present absorber to be optimum, a larger damping ratio is gotten. Overall, the present absorber gives the excellent vibration suppression as evidenced by the less seven parameters: *A*_1_(*g*_*S*_), *A*_1_(*g*_*T*_), *A*_1_(*f*_opt_), *A*_1_(*ξ*), *A*_1_(*g*_c1_), *A*_1_(*g*_c2_) and *A*_1_(*g*_t_). The characteristic at the anti-resonance forced angular frequency ratio of the present absorber can’t notably decrease because of the increase in damping ratio, viz, near *g* = *f*_opt_, a little drop still exists (2.7449 < 2.7949).

The mass-spring main vibration system consists of a cantilever beam whose main mass *m*_1_ = 4.5 kg and main stiffness *k*_1_ = 18071 N/m. The mass-spring-damper dynamic vibration absorber, whose attached mass *m*_2_ = 0.45 kg, is installed at the end of the mass-spring main vibration system. The amplitude of the pulsating force acting on the mass-spring main vibration system is *F* = 4.4 N.

**Fig 8:**
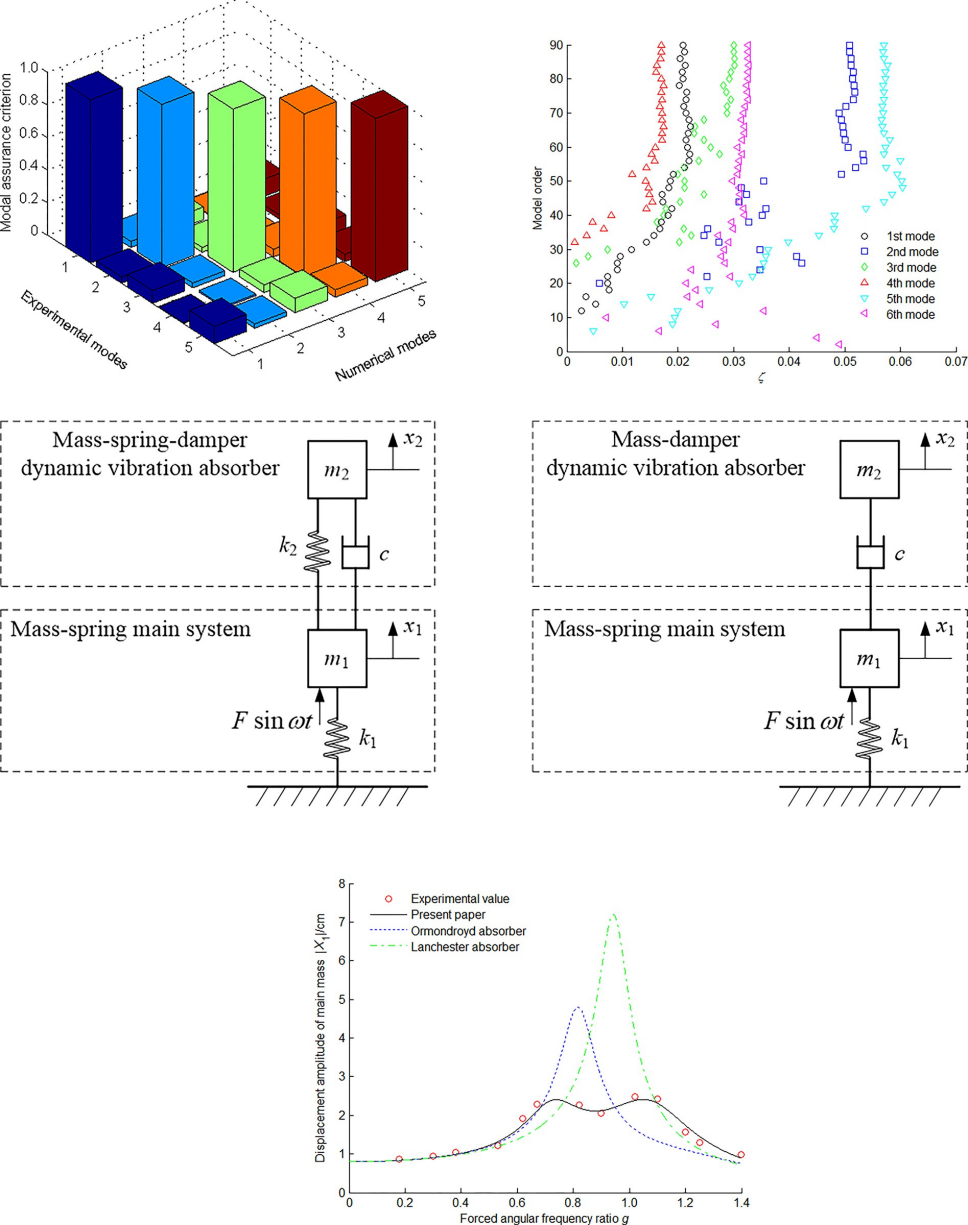
Experimental results. (a) Modal assurance criterion. (b) Experimental value of damping ratio. (c) The Ormondroyd absorber. (d) The Lanchester absorber. (e) Amplitude-frequency response characteristic of main mass.

However, adding a dynamic vibration absorber to an original one-degree-of-freedom vibration system results in a new two-degree-of-freedom vibration system. The experimental results are shown in Fig 8. In order to degrade the random error and improve the signal to noise ratio, the mean results of five times about the firing experiment are employed. The modal assurance criterion between numerical and experimental modes is illustrated in Fig 8A. There are five characteristic vectors. Everyone is 630×1 column vector forming 630×5 vibration shape matrix. The modal assurance criterion is a pure mathematical criterion for checking the consistency between two eigenvectors. The perfect correlated mode shapes are in an appropriate order. If the modes are evidently separated according to the available sparse spatial information and the measurement noise is negligible, those are assigned with high reliability. It is seen from in Fig 8B that the damping ratios of the first, third and fourth mode shapes relatively concentrates. Generally speaking, the high-order modal damping ratio is relatively larger, therefore their vibration response components can rapidly attenuate. It is seen from in Fig 8E that the numerical simulation results of the present paper’s absorber are closer to the vibrational experimental results than those of the Ormondroyd absorber and Lanchester absorber. Moreover, the present paper’s absorber has larger band width frequency *ω*_b_ than the Ormondroyd absorber and Lanchester absorber. When these theoretical findings are applied to real-world scenarios, there are the following limitations or potential inaccuracies: replacing the distributed mass with the concentrated mass, air damping force, and the experimental testing displacement amplitude of main mass.

## 6. Conclusions

1. A general method of obtaining the explicit exact solution to the optimum damping ratio was presented to improve the accuracy of computing the dynamic vibration absorber’s optimum parameters. The attached mass was also proposed in consistency with the main mass. Some exact analytic expressions of displacement amplitude gain, and fixed point coordinate were attained according to the real number form of differential equation of load motion. The most important job in this manuscript is to get the optimum damping ratio of the dynamic vibration absorber utilizing L’Hospital first rule.
2. Some computational parameters of the main vibration system were attained. The role and effect of the dynamic vibration absorber were analyzed through comparing those of other absorbers.
3. To one’s regret, many present research results thought that the maximum value of the fixed point of the main vibration system was gotten. In reality, the fixed point theory can only obtain the extreme large value, not by any means the maximum value.
4. The construction of the general method using L’Hospital first rule may give a theoretical base for obtaining the accurate optimum parameter of the dynamic vibration absorber. The general method in the manuscript is appropriate to other complex dynamic vibration absorbers.
5. The presented study can be expanded to systems with more degrees of freedom through the following methods: the main system with damping element, the dynamic vibration absorber adopting the spring and damping elements in series, and using the distributed mass.

At last the formulae given here pave another road toward the goal of constituting a stable foundation stone for accurately predicting the dynamic performances of the main vibration system to be expected from some given known parameters, further for optimally “designing” the required efficient dynamic vibration absorber, fully without being confined to empiricism formula and experimental data elsewhere.

## References

[1] Stephen Timoshenko. Vibration problems in engineering. Second edition—Fifth printing, D. Van Nostrand Company, Inc., New York, 1946: 240–252.

[2] Pieter Den Hartog Jacob. Mechanical vibrations. Fourth edition, Dover Publications, Inc., New York, 2016: 87–106.

[3] Erich Hahnkamm. Die dämpfung von fundamentschwingungen bei veränderlicher erregerfrequenz. Ingenieur-Archiv, 1933, 4(2): 192–201 in German.

[4] Brock John E. A note on the damped vibration absorber. Journal of Applied Mechanics, 1946, 13(4): A–284.

[5] Brock John E. Author’s correction. Journal of Applied Mechanics, 1947, 14 (1): A–80.

[6] Ye Yu, Xiu-xian Jia, Huajiang Ouyang, Yu Du, Yiqiang Peng. Dynamic properties investigation of an acoustic black hole beam with dynamic vibration absorber based on analytical method. Journal of Sound and Vibration, 2024, 570: 118053.

[7] Xing Zhao-Yang Yang Xiao-Dong. A combined vibration isolation system with quasi-zero stiffness and dynamic vibration absorber. International Journal of Mechanical Sciences, 2023, 256: 108508.

[8] Yuan Du, Tongda Zou, Fuzhen Pang, Chao Hu, Yong Ma, Haichao Li. Design method for distributed dynamic vibration absorbers of stiffened plate under different boundary constraints. Thin-Walled Structures, 2023, 185: 110494.

[9] Hui Sheng, He Meng-Xin Ding Qian. Vibration suppression by mistuning acoustic black hole dynamic vibration absorbers. Journal of Sound and Vibration, 2023, 542: 117370.

[10] Qiang Wang, Jiaxi Zhou, Kai Wang, Jinghang Gao, Qida Lin, Yaopeng Chang, Daolin Xu, Guilin Wen. Dual-function quasi-zero-stiffness dynamic vibration absorber: low-frequency vibration mitigation and energy harvesting. Applied Mathematical Modelling, 2023, 116: 636–654.

[11] Xiaoyu Chen, Yonggang Leng, Fei Sun, Xukun Su, Shuailing Sun, Junjie Xu. A novel triple-magnet magnetic suspension dynamic vibration absorber. Journal of Sound and Vibration, 2023, 546: 117483.

[12] Yi Qin, Tan Jin Jack Hornikx Maarten. Application of multiple dynamic vibration absorbers in reducing low-frequency vibration of a floor-like lightweight joist structure: comparison of experimental and computational results. Applied Acoustics, 2023, 211: 109437.

[13] Shaoqing Wu, Hangxing Li. A data-driven design method of distributed dynamic vibration absorber for broadband vibration suppression of thin-walled structures. Thin-Walled Structures, 2023, 182: 110264.

[14] Yaopeng Chang, Jiaxi Zhou, Kai Wang, Daolin Xu. Theoretical and experimental investigations on semi-active quasi-zero-stiffness dynamic vibration absorber. International Journal of Mechanical Sciences, 2022, 214: 106892.

[15] Dawei Li, Kohju Ikago, Ao Yin. Structural dynamic vibration absorber using a tuned inerter eddy current damper. Mechanical Systems and Signal Processing, 2023, 186: 109915.

[16] Ruqi Sun, Waion Wong, Li Cheng. Optimal design of a tunable electromagnetic shunt damper for dynamic vibration absorber. Mechatronics, 2022, 83: 102763.

[17] Zhiwei Su, Zhiwei Zheng, Xiuchang Huang, Hongxing Hua. Research on dynamic vibration absorber with negative stiffness for controlling longitudinal vibration of propulsion shafting system. Ocean Engineering, 2022, 264: 112375.

[18] Siqi Lin, Yongshan Zhang, Yingjing Liang, Yijie Liu, Chunming Liu, Zhiyong Yang. Bandgap characteristics and wave attenuation of metamaterials based on negative-stiffness dynamic vibration absorbers. Journal of Sound and Vibration, 2021, 502: 116088.

[19] Hangxing Li, Shaoqing Wu, Qiang Chen, Qingguo Fei. Design of dynamic absorbers to control the flexural resonant vibration of structures characterized by multiple natural modes. Journal of Sound and Vibration, 2021, 513: 116415.

[20] Mohammed Kassem, Zhichun Yang, Yingsong Gu, Wei Wang, Ehab Safwat. Active dynamic vibration absorber for flutter suppression. Journal of Sound and Vibration, 2020, 469: 115110.

[21] Behshad Noori, Robert Arcos, Arnau Clot, Jordi Romeu. Control of ground-borne underground railway-induced vibration from double-deck tunnel infrastructures by means of dynamic vibration absorbers. Journal of Sound and Vibration, 2019, 461: 114914.

[22] Beltrán-Carbajal F, Silva-Navarro G. Active vibration control in Duffing mechanical systems using dynamic vibration absorbers. Journal of Sound and Vibration, 2014, 333: 3019–3030.

[23] Chegury Viana Felipe Antonio, Iamin Kotinda Giovanni, Alves Rade Domingos, Valder Steffen Jr. Tuning dynamic vibration absorbers by using ant colony optimization. Computers and Structures, 2008, 86(13–14): 1539–1549. doi: 10.1016/j.compstruc.2007.05.009

[24] Krzysztof Kecik. Assessment of energy harvesting and vibration mitigation of a pendulum dynamic absorber. Mechanical Systems and Signal Processing, 2018, 106: 198–209. doi: 10.1016/j.ymssp.2017.12.028

[25] Kefu Liu, Jie Liu. The damped dynamic vibration absorbers: revisited and new result. Journal of Sound and Vibration, 2005, 284(3–5): 1181–1189.

[26] Ren M Z. A variant design of the dynamic vibration absorber. Journal of Sound and Vibration, 2001, 245(4): 762–770.

